# Theoretical care model for children with congenital Zika virus syndrome in the family context*

**DOI:** 10.1590/1518-8345.4057.3458

**Published:** 2021-06-28

**Authors:** Gracimary Alves Teixeira, Aylla Nauana da Silva, Larissa Soares Mariz Vilar de Miranda, Marcela Paulino Moreira da Silva, Elisângela Franco de Oliveira Cavalcante, Bertha Cruz Enders

**Affiliations:** 1 Universidade Federal do Rio Grande do Norte, Escola de Saúde, Natal, RN, Brazil.; 3 Universidade Federal do Rio Grande do Norte, Departamento de Enfermagem, Natal, RN, Brazil.; 5 Universidade Federal de Campina Grande, Departamento de Enfermagem, Campina Grande, PB, Brazil.

**Keywords:** Microcephaly, Zika Virus Infection, Family Relations, Mothers, Family, Grounded Theory, Microcefalia, Infecção pelo Zika Vírus, Relações Familiares, Mães, Família, Teoria Fundamentada, Microcefalia, Infección por el Virus Zika, Relaciones Familiares, Madres, Familia, Teoria Fundamentada

## Abstract

**Objective:**

to develop a theoretical model about the care of children with Congenital Zika Virus Syndrome in the family context.

**Method:**

the Straussian Grounded Theory and the theoretical/philosophical framework of Callista Roy and Leonardo Boff were used. It was carried out in northeastern Brazil, with 19 participants, in four sample groups. The data were collected and analyzed simultaneously, using the constant comparison method.

**Results:**

the theoretical model comprising the mother’s care for the child with Congenital Zika Virus Syndrome in the family context is formed by five categories: revealing family care, centered on the mother, to the child with the syndrome; identifying the maternal bond that determines the care for children with the syndrome in the family context; identifying the factors that hinder the mother’s care for the child with the syndrome; recognizing the evolution of the child with the syndrome in the face of early stimulation care; and recognizing harms due to the absence of early stimulus to children with the syndrome.

**Conclusion:**

the phenomenon runs through the care of the child with Congenital Zika Virus Syndrome, in the family context and centered on the mother, and is faced with the need for shared responsibility among the family members.

## Abstract (pt)

**Objetivo:**

elaborar um modelo teórico acerca do cuidado à criança com Síndrome Congênita pelo Zika vírus no contexto familiar.

**Método:**

utilizou-se a Teoria Fundamentada nos Dados Straussiana, e referencial teórico/filosófico de Callista Roy e Leonardo Boff. Foi realizada no nordeste brasileiro, com 19 participantes, em quatro grupos amostrais. Os dados foram coletados e analisados simultaneamente, pelo método de comparação constante.

**Resultados:**

o modelo teórico compreendendo o cuidado da mãe à criança com Síndrome Congênita pelo Zika vírus no seu contexto familiar é formado por cinco categorias: revelando o cuidado da família, de forma centrada na mãe, à criança com a síndrome; identificando a vinculação materna que determina o cuidado à criança com a síndrome no contexto familiar; identificando os fatores que dificultam o cuidado da mãe à criança com a síndrome; reconhecendo a evolução da criança com a síndrome diante do cuidado de estimulação precoce; reconhecendo prejuízos em virtude da ausência do estímulo precoce à criança com a síndrome.

**Conclusão:**

o fenômeno perpassa pelo cuidado à criança com Síndrome Congênita pelo Zika vírus, no contexto familiar, centrado na mãe e se depara com a necessidade de responsabilidade compartilhada entre os membros da família.

## Abstract (es)

**Objetivo:**

desarrollar un modelo teórico sobre el cuidado de niños con Síndrome Congénito por el Virus del Zika en el contexto familiar.

**Método:**

Se utilizó la Teoría Fundamentada en los Datos de Strauss y el marco teórico/filosófico de Callista Roy y Leonardo Boff. Se llevó a cabo en el noreste de Brasil, con 19 participantes, en cuatro grupos de muestra. Los datos fueron recolectados y analizados simultáneamente, utilizando el método de comparación constante.

**Resultados:**

el modelo teórico que comprende el cuidado de los niños con Síndrome Congénito por el Virus del Zika por parte de la madre en su contexto familiar está conformado por cinco categorías: revelar el cuidado familiar del niño con el síndrome, centrado en la madre; identificar el vínculo materno que determina el cuidado del niño con síndrome en el contexto familiar; identificar los factores que dificultan el cuidado materno del niño con el síndrome; reconocer la evolución del niño con el síndrome frente a los cuidados de estimulación temprana; reconocer daños causados por falta de estimulación temprana a los niños con el síndrome.

**Conclusión:**

el fenómeno pasa por el cuidado del niño con Síndrome Congénito por el Virus del Zika, en el contexto familiar, centrado en la madre y se enfrenta a la necesidad de corresponsabilidad entre familiares.

## Introduction

In 2015, an abnormal increase in children with microcephaly associated with the Zika virus recently arrived in Brazil began in the country^(^ 1 ^-^ 3 ^)^. Microcephaly is a congenital anomaly in which the Cephalic Perimeter (CP) of the child born at term is equal to or less than 32 cm^(^ 4 ^)^. With the advancement of cases in 2016 and of research studies on the phenomenon among newborns, it was observed that in vertical transmission of the virus there were also other malformations in addition to microcephaly, known as Congenital Zika Virus Syndrome (CZVS)^(^ 5 ^)^.

CZVS is a congenital anomaly associated with Zika virus infection, characterized by intracranial calcifications; decreased brain volume; severe brain abnormalities; ocular and auditory anomalies; delay in cognitive, motor and speech development; cerebral palsy; epilepsy; dysphagia; irritability, that is, the syndrome can manifest itself through a wide range of abnormalities, in addition to the reduced head circumference^(^ 6 ^-^ 8 ^)^.

It is a complex anomaly with a marked impact on the child’s health and growth and development, family life and health services, due to the complexity of care that the condition demands. In addition, uncertainties about functional limitations, future implications and demands for care to come, still under study^(^ 9 ^)^.

Due to the great possibility of severe limitations of cognition and motor skills, there is a need for coordinated and family-centered health care^(^ 9 ^-^ 10 ^)^. There is evidence that family-centered care for children with special needs has better results^(^ 11 ^)^.

In this perspective, care for children with CZVS must be multi-professional to support the family in comprehensive care for the child’s biological, psychological and social development. In addition to the daily family care to meet their needs related to functional limitations, it includes early stimulation in the family care routine^(^ 10 ^,^ 12 ^)^. Early stimulation, especially in the first years of life, a period in which brain development occurs more quickly, maximizes the potential for physical growth and neurological, behavioral, cognitive, affective and social maturation^(^ 12 ^)^.

Therefore, there is a need for the Family Health Strategy (FHS) team to offer care that supports the family and provides the child with early stimulation, in their family life, according to the guidance of the specialized team that assists the child. This care developed by the family is essential, as the team of specialized professionals stimulates the child’s growth and development, but requires continuity by the family in their daily lives, for the child’s good evolution.

However, there is little action by the FHS professionals, including the Nurse, due to the lack of knowledge of the appropriate actions to assist children with CZVS, in the home context, with the involvement of other professionals in this care practice being better known^(^ 1 ^,^ 10 ^)^.

Although some studies address children with CZVS and its repercussions on family dynamics, they are presented in a descriptive way^(^ 1 ^,^ 10 ^,^ 13). The care of children with CZVS, in the family context, as a process in the context of Nursing in primary health care, has been little studied. This gap can be related to the fact that the first cases of CZVS, worldwide, appeared in Brazil in 2015^(^ 4 ^,^ 7 ^)^ and, therefore, theoretical models that explain the process and concepts of child care with CZVS in the family context may still be in the process of development.

Thus, this study aims to develop a theorization of care for children with CZVS in the family context, and its concepts for guiding health professionals about this care. It is expected that the concepts can be used by the health disciplines, as well as that of Nursing and that, in this way, it is possible to guide assistance in the multi-professional care process. Thus, the objective of this study is to elaborate a theoretical model about care for children with Congenital Zika Virus Syndrome in the family context.

## Method

This is a qualitative approach study, guided by the Straussian Grounded Theory (GT). This GT method provides a systematic means of collecting data to describe, explain and predict the professional practice^(^ 14 ^)^.

Grounded theory means: “The theory was derived from data, systematically gathered and analyzed through a research process”, that is, the researcher starts with an area of study and allows the theory to emerge from the data. Thus, the theory derived from the data tends to offer a better understanding - and provide a guide for action in practice - than the theories derived from the gathering of concepts, through speculation or experience, because the theory derived from the data comes close to the reality experienced by the participants^(^ 14 ^)^.

The study was carried out in a Child Rehabilitation Center (CRC), located in a capital city in northeastern Brazil. It is an organ belonging to the State Department of Public Health and provides assistance to people with any type of disability, be it physical, mental, sensory and/or multiple, aged 0 to 18 years old. The institution was chosen because it is a reference center in the state capital for assistance to children with CZVS, as a strategy to identify the families of children with this diagnosis and the approach to participate in the study.

The selection of participants followed these inclusion criteria: family members who provided direct care to children with CZVS; aged ≥ 1 year old and with a minimum time of six months living with the child with CZVS. The exclusion criteria were family members whose children were diagnosed with malformations other than CZVS, family members without cognitive conditions to conduct the conversation, and members of families where the children died.

The study then had the participation of 19 informants who experience the care of children with CZVS. Four sample groups were formed: Group I, the largest, with twelve mothers; Group II was composed of three parents; Group III by two CRC physiotherapists, and Group IV by two FHS nurses. The data collected determined the need to form sample groups in addition to those composed by family members due to the questions raised during the collection^(^ 14 ^)^. The groups were formed according to the hypotheses about care that were raised during the interviews and needed to be verified.

The determination of the number of informants in each group followed the principle of theoretical saturation; that is, as collection took place, data was analyzed simultaneously, in order to identify the properties (characteristics of a category) and dimensions (similarities and differences), until no new properties and dimensions appeared in the data that characterized saturation.

Data collection took place from April to October 2018, through a recorded interview held in a private location and chosen by the informant. The initial interviews took place predominantly at the CRC and some at the informants’ homes. In the interviews with the mothers, a script was used to request sociodemographic information and to discuss the care provided to children with CZVS, through the following guidance: “I would like you to tell me how it has been for you to take care of your child with this problem”.

As the interviews took place, scientific initiation scholarship holders trained in audio transcription transcribed the recordings and the main researcher checked the transcription and started analyzing the data before proceeding to the next interview.

Furthermore, observation and memos were part of data collection, simultaneously with the interview. The memo consists of recording what was viewed and interpreted by the researcher during the interviews^(^ 12 ^)^. In this study, the observations of shyness, sadness, crying, as well as the reflections and questions raised by the researcher that emerged during the study, were recorded in the memos. Among the records of the memos were the questions that emerged during collection and comparative analysis of the data and helped in the development of the categories, or concepts, and their dimensions. The records contain points about care that should be clarified in the course of the study, for example: who is the caregiver and how is the care of the child’s daily life, what therapeutic actions are carried out, which health professionals are involved in the care, and feelings of the caregivers, among others.

The questions that emerged from data analysis also helped to define the sample groups. The fact that the first sample group was formed by the mothers was due to the fact that they were the main companions of the children to specialized care in the reference service. With data analysis new questions arose, such as: How do parents participate in the care of children with CZVS? How do parents feel about the responsibility of caring for children with CZVS? Thus, the need to compare the data of the maternal interviews with the parents’ experience gave rise to the second sample group. Likewise, the information reported by the mothers about the involvement of professionals in care raised questions about the way in which professional/family care occurs and that need to be clarified: How do CRC physiotherapists promote early stimulation of children with CZVS? How nurses carry out the growth and development consultation of children with CZVS?

In view of these questions, individual interviews took place with all respondents from the other sample groups, in private environments. Those carried out with physiotherapists and nurses took place in their workplaces, as indicated by the mothers and prior appointment. The physiotherapists participating in this study acted in the early stimulation of children’s growth and development in the CRC. Nurses assisted these children in the FHS from the first days of life, developing care aimed at monitoring growth and development. The interviews carried out with the parents were scheduled in the respective households due to the difficulty of finding them in the health services. The mothers were interviewed at the reference service, according to their availability.

Data analysis was carried out using comparative analysis concurrently with data collection, employing open, axial and selective coding procedures, processes that allow for the construction of categories and subcategories, demonstrating the concepts and dimensions elaborated from the data, according to Strauss and Corbin. These methodological stages are permeated by comings and goings, indicating the necessary process to relate concepts and categorize the data in the definition of the central category. The central category explains the phenomenon under study, that is, it is the interpretation of the research theme^(^ 14 ^)^.

In open coding, the concepts were identified, from the data, with their properties and dimensions. Subsequently, the differences and similarities between the data already coded from previous interviews were verified and grouped with the provisional concepts. This coding process allowed for the implementation of the axial coding stage, where the process of grouping fragmented data during open coding began. And throughout this regrouping process, the stage of selective codification developed in which the categories were refined with more abstract concepts, integrating the subcategories originated from the concepts/codes arising from the interviews, giving rise to the central category. Thus, the theoretical model was formed with the categories/components of actions-interactions, conditions and consequences.

In the construction process, Leonardo Boff’s philosophical perspective on care was used to position the author’s understanding of the affective aspect of the family/child/care relationship with CZKS. In this perspective, care is an attitude much more than an act, as it represents an occupation, concern; it demands accountability and requires affective involvement with the other^(^ 15 ^)^. Roy’s Adaptation model served to visualize children with CZKS as holistic beings, and their families undertaking the adaptation path affected by the stimuli of life, as a result of anomalies and the involvement of caregivers in the search for adaptive capacity^(^ 16 ^)^.

The research respected the ethical principles, following the favorable opinion of the Ethics and Research Committee (*Comitê de Ética e Pesquisa*, CEP) of the Federal University of Rio Grande do Norte, of 07/14/2017, with CAAE No. 69859717.1.0000.5537. And to maintain the anonymity of the participants, they were listed according to the order of interviews and sample group.

## Results

The families of these children with CZVS live in the inland or on the outskirts of a capital city in northeastern Brazil, with difficulty in transport to the child care reference services. The mean family income is nearly 1 minimum wage, coming from sickness benefit, since child care requires full attention from mothers, which makes maternal return to the labor market unfeasible. In addition to these conditions of vulnerability, there is the low level of schooling of these families.

The meanings of caring for children with CZVS in the family context constructed in this study were carried out based on the concepts raised from the data of the experiences, perceptions and feelings of mothers and fathers of children with CZVS about care within the family. This allowed describing the occurrence of the phenomenon and the schematic demonstration of its structure and organization of the connections as a process.

Thus, during the process of data analysis at different levels, open, axial and selective, the discovery of the three components of the theoretical model occurred: actions-interactions; conditions and consequences. The relationships between the data of these elements appear as categories and subcategories, each containing its own elements and dimensions that identify it. Together, these structural elements describe and explain the process of the phenomenon addressed.

The structure of the theoretical model elaborated in this study is represented in Figure 1, with the five categories and their respective subcategories that support the central category of the phenomenon: Understanding mother’s care for children with CZVS in the family context. In this figure, it is shown that the central category identifies the mother as the main protagonist member of the care for children with CZVS in the family. Although it receives some support from the father, it is the mother who attends, with selflessness, to the demands of daily care, while experiencing feelings of overload when facing difficulties and the absence of support from others.

**Figure 1:** Components of the theoretical model: Understanding mother's care for children with CZVS in the family context, and the categories and subcategories that integrate it. Natal, RN, Brazil, 2018

**CENTRAL CATEGORY: Understanding mother's care for children with CZVS in the family context.**		**CATEGORIES**	**SUBCATEGORIES**
	**ACTIONS-INTERACTIONS**	- Revealing family care, focused on the mother, to the child with CZVS*.	- Demanding intensification of the mother's attention, for the care of the child with CZVS* in the family context; - Caring for the child with CZVS* centered on the mother with the support/help between family members; - Experiencing maternal care in the basic and clinical/specific needs of children with CZVS*; - Stimulating the growth and development of the child with CZVS* in family life; - Stimulating the growth and development of the child with CZVS* through professional support;
	**CONDITIONS**	- Identifying the maternal bond that determines the care of the child with CZVS* in family life; - Identifying the factors that hinder the care of the mother to the child with CZVS*.	- Materializing unconditional involvement (love) between mother and child with CZVS*; - Experiencing the mother's confidence/distrust in leaving the child with CZVS* to the care of family members; - Facing feelings of loneliness, maternal exhaustion and acceptance of the health condition of the child with CZVS*; - Having a fragile support network for maternal care for children with CZVS*; - Reducing the mother's time in stimulating the growth and development of the child with CZVS* due to maternal tiredness and exhaustion; - Experiencing the difficulty of access to assistance that stimulates the growth and development of children with CZVS*.
	**CONSEQUENCES**	- Recognizing the evolution of the child with CZVS* in view of early stimulation care; - Recognizing losses due to the lack of early stimulation to children with CZVS*.	- Perceiving the evolution in the development of the child with CZVS* with the stimuli performed; - Perceiving the delay in the development of the skills of the child with CZVS* without the assistance of early stimulation.

* CZVS = Congenital Zika Virus Syndrome

This figure was validated. In the validation stage, the illustration of the model and its synthesis were presented to the mothers of children with CZVS, who accompany their children in therapy at the CRC, and who recognized and reiterated the categories and subcategories of the theoretical model: *Understanding mother’s care for children with CZVS in the family* context.

For the presentation of the theoretical model, an illustrative figure was constructed about the care for children with CZVS, in their family context, centered on the mother, although the families of these children are composed of father, mother and siblings. In this model there is the presence of the father; however, only as an aid, especially in playing with children at times when there are no professional and personal commitments. Therefore, the mother is the one who takes responsibility for all the care for the child with CZVS and full time, as the father does not feel responsible for this care. He helps when it suits him, as a father explains: *When the mother is at home, as a father I do not worry. Only when the mother leaves home, do I as a father worry and try to do as much as possible. I see the mother caring and I care the way she does (Father 03)*.

**Figure 2:**
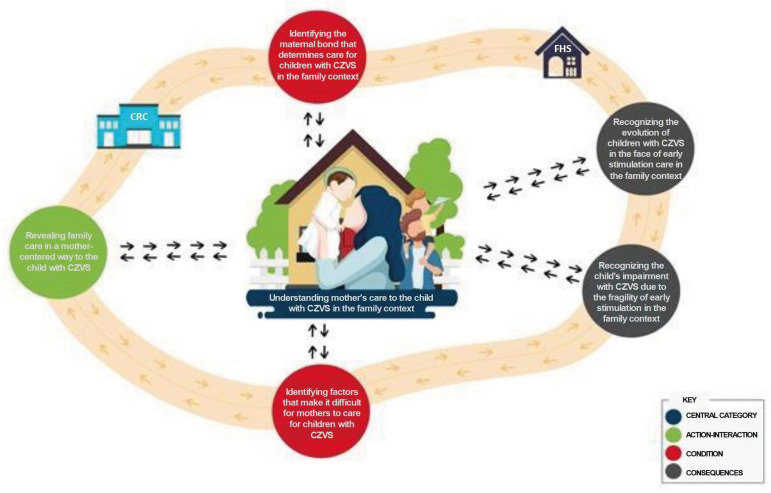
Theoretical model comprising the mother’s care for children with CZVS in their family context CZVS = Congenital Zika Virus Syndrome; CRC = Child Rehabilitation Center; FHS = Family Health Strategy

Thus, the family, especially the mother, is the one who observes, identifies and seeks to meet the needs of the child with CZVS. These children demand routine care (food, hygiene) common to every child; specific care for situations of irritability, seizures, changes in sleep, physical and emotional dependence; and care for the early stimulation of growth and development.

Therefore, the reception and resolution of the needs of children with CZVS depend on the mothers, with sporadic help, for some care, on the child’s parents and siblings. Often, the care of family members is reduced to playing, for a few moments, while the mother provides the child’s bath and food, and cares for the home.

*Mothers are overwhelmed by everything: it is for care, it is for taking care of children, at home. Not because of the boy, because everywhere I go he is welcome, even my family calls me to bring him, but overload of responsibility in caring for the child. The father spends the day working, only goes to him once lost, once in a blue moon (Mother 12).*

As noted in this previous statement, it is reinforced that the responsibility for the care of children with CZVS lies in the mothers, as the father places himself as the provider of the home and the mother’s helper, not feeling responsible for the care.

Therefore, both as a result of the weakened support network, as well as the child’s emotional and physical dependence on the mother, and the non-verbal communication understood almost exclusively by the mother, maternal tiredness and exhaustion consequently occur because, in addition to the usual care to a standard child, these children in study demand intensification of the mother’s attention to understand and meet their needs.

*The bath has to be more delicate because she is soft; the question of food has to be in the correct posture because she choked a lot; the issue of sleep also that she has reflux, she has to be always attentive because she wakes up coughing, trying to choke. Because they are so limited, they are very delicate care, we dedicate ourselves more deeply (Mother 09).*

With these different needs, they demand the presence of the mothers for the care of the child by family members, or the mothers can only trust and feel safe to be absent from the residence, for a moment of need, according to the development of the child that allows for less dependence.

Given these factors that cause the maternal burden of care for the home and the child, there is a reduction in the mother’s time for the early stimulation of the growth and development of the child with CZVS, causing a reduction in the quality of life of both.

In this direction, mothers, even with the losses in their self-care, social and work renouncements, and physical and mental fatigue, face this burden of responsibility and care due to the involvement and unconditional love between mother and child with CZVS. This is because there is an integral involvement between the mother-child binomial in which the two come together in full, expanding maternal limits and differentiating themselves from all other family members. As we can see below from the participant’s speech: *today she is everything to me, I can’t talk, both she and I are everything to each other! I am the legs, the arms, I am the eyes, I am all of her and she is very much to me, even with limitations, with her way. As I have another child to like and love is the same, but the greatest care is with her, because as she doesn’t know anything alone, I am the head, I am the arms, I am the legs, I am all hers (Mother 10).*

In addition, the mother has a resilient capacity for the hope that with her care the child with CZVS will develop and walk. And regarding this motivation, the mothers have reported the participation of CRC physical therapists and FHS nurses as supporters in the early stimulation of the growth and development of children with CZVS.

The physiotherapists encourage and demonstrate the exercises to be performed by the family at home, and the FHS nurses during consultations to monitor growth and development reinforce the importance of continuing this stimulation by the family in the daily life of the child with CZVS.

Therefore, children who are inserted in the family environment, and are simultaneously assisted by the specialized team that encourages the continuation of early stimulation in family life, and in turn, receive stimulation care by the family at their home, present a better evolution of the capabilities of their existing potentialities. *Lying on the floor, we kept making movement on the arm and leg. In a 1,000 liter water tank, her father puts water and stays inside with her playing. On the bed he is lifting her arms, her legs, capsizing this way and that, playing with her. And with the brushes, that her little hand was closed, going over the hand so that she would feel itchy and try to open her hand. We would take a diaper and put it under her arm, hold it for her to try to walk, if she couldn’t we would firm her foot and start talking, she would look at us laughing and trying. Thank God today she is walking normally (Mother 05).*

However, there is a delay in the development of the skills of children with CZVS, as they have difficulties in accessing specialized assistance that promotes a routine of early stimulation of their growth and development to be performed routinely by their family.

## Discussion

The families of the participating children with CZVS live in the inland or on the outskirts of the city where the study was conducted and reported difficulty in transport to the child care reference services. This problem reflects the inequalities and difficulties in accessing health services in Brazil by children with disabilities^(^ 17 ^)^, and the problems faced by family caregivers, due to institutional policies that hinder service coordination and communication^(^ 18 ^)^. Despite the strengthening of the Brazilian health services by the federal government, for the health care of children with CZVS, the difficulty of access to specialized services of the Unified Health System (*Sistema Único de Saúde*, SUS) and care fragmentation continue^(^ 17 ^)^.

Poverty is another factor that affects the accessibility and equality of the services^(^ 10 ^,^ 17 ^)^. In the study, the family income of the families is nearly 1 minimum wage, from sickness benefit - Continuous Benefit (*Benefício de Prestação Continuada*, BPC) - because child care requires full attention from the mothers and, therefore, they do not return to the labor market. In addition to these vulnerability factors, there is the low level of schooling of the families.

Thus, mothers of children with CZVS face daily difficulties and challenges in caring for children with changes in their development and growth. Based on this premise, the categories and subcategories emerging from the data describe and explain in an interrelated way the care for children with CZVS in the family context. With this, the meanings originated guide the care by the health professionals, especially nurses from the FHS, on the promotion of strengthening the maternal support network and sharing of care.

It is noticed that the care for children with CZVS in the family context, centered on the mothers, causes maternal overload that, when positioning themselves as the core of the care process, full time, causes harms to their quality of life. With the attributions of childcare inherent to women, due to gender inequalities, the resulting consequences are fatigue, exhaustion and insufficient time for self-care, as well as for early stimulation of the growth and development of children with CZVS. Thus, a study that aimed to examine how parents experience their work as caregivers for their child with chronic disability reinforces the mother’s hegemony and significant daily commitment^(^ 19 ^)^. The tasks are mainly performed by them, with occasional support from other women in the family, such as grandmothers, while the fathers carry out logistical activities and play with the children^(^ 20 ^)^.

According to the Brazilian Ministry of Health, early stimulation of the child’s growth and development is essential during the first 3 years of life, due to the period in which neurological, behavioral, cognitive, affective and social maturation occurs^(^ 12 ^)^. However, it is observed in the data that, with the overload of maternal care, it has not been possible to provide the time necessary for early stimulation in the family.

Therefore, it is essential to have a professional eye for the care of the children and their families, analyzing the family structure as a whole: which members make up this family, its context, the affective bonds involved and the interaction between the individuals, the roles played by each family member, as well as understanding the reorganization of the families in the face of birth and the needs of children with CZVS^(^ 21 ^)^.

According to Sister Callista Roy and the Adaptation model, the stimulus generated by the family, social and environmental environment is the best way to promote development and adaptations in children^(^ 16 ^)^. In spite of this, from the data of this study it was possible to realize that access to specialized assistance for children with CZVS is as deficient as the early stimulation of these children at home.

In this context, the nurse, as a FHS professional, who observes the child with CZVS at home, must work with families in order to promote the family and social insertion of these children, enhancing their development, as well as strengthening the network support and shared care.

Furthermore, caring for people is the way to do Nursing^(^ 22 ^)^. When referring to care, the act of caring is passed through and refers to an attitude, which means an emotional responsibility with the other or a form of complacency and service^(^ 15 ^)^. Thus, the concept of family care is expanded, which requires involvement, responsibility and a sense of competence to develop the child’s skills from everyone, whether parents, mothers, uncles, aunts, grandfathers, and grandmothers.

In such a way, a study points out that the insertion of the father in prenatal, delivery and postpartum care, as a participant in the process, contributes to the reconstruction of the male identity of home provider and support of the mother to become part of the mother-family-baby triad, because man’s participation in the process of gestating and giving birth promotes involvement, bonding and accountability in the care of the child^(^ 23 ^)^.

In addition to the daily family care to meet the basic needs inherent to childhood, children with CZVS have care related to functional limitations, and the need for early stimulation in the family care routine. Thus, a study carried out in Canada with families of children with chronic diseases and/or disabilities is similar to the need for children with CZVS, for full-time family care. However, that same study in Canada differs in that it presents parental participation in sharing responsibility for the care of children with chronic diseases and/or disabilities^(^ 24 ^)^.

In this sense, the Nursing professional, by sensitizing family members to share care with the mother, making the form of concern and care as shared among family members, allows the mother (caregiver) to reduce her burden and enable maternal self-care, as well as the early stimulation of the child with CZVS, in view of the need to develop their skills.

The research, even following all the methodological rigor, presents as a limitation the fact of addressing the experience of a single scenario. Thus, new studies are suggested that come to explore the contexts in other services. In addition, it is recommended to carry out further studies that may include children who are absent from specialized care services for several reasons, so as to include other possibilities of caring for children with CZVS in the family context.

Therefore, it is expected that this study will contribute to the disciplines of health and the science of Nursing, in the wake of the formulation of public policies and expansion of discussions between managers and health professionals, especially in Nursing, to qualify the health team in the care process that seeks to promote the strengthening of the support network and to raise awareness of the need for accountability for shared care among the family members. Co-management with the family, especially with the maternal figure, in the construction of knowledge and actions, enhances active participation and guides specific behaviors in the therapeutic process of children with CZVS.

## Conclusion

The phenomenon permeates the care for children with CZVS in the family context, centered on the mother, and is faced with the need for shared responsibility among the family members. In addition to that, the theoretical model that explained the phenomenon of *Understanding mother’s care for children with CZVS in their family context* guides Nursing care in this process, by strengthening the support network for the care of these children and raising awareness of the need to reconstruct male identity.
